# Sensor-Embedded Automatic Grasping Forceps for Precise Corneal Suture in Penetrating Keratoplasty

**DOI:** 10.3390/mi12050484

**Published:** 2021-04-23

**Authors:** Hyung-Gon Shin, Ikjong Park, Keehoon Kim, Hong-Kyun Kim, Wan-Kyun Chung

**Affiliations:** 1 Department of Mechanical Engineering, Pohang University of Science and Technology, Pohang 37673, Korea; ko.h.g.shin@gmail.com (H.-G.S.); too1213@postech.ac.kr (I.P.); khk@postech.ac.kr (K.K.); 2 Department of Ophthalmology, School of Medicine, Kyungpook National University, Daegu 41944, Korea; okeye@knu.ac.kr; 3 Institute for Convergence Research and Education in Advanced Technology, Yonsei University, Seoul 03722, Korea

**Keywords:** medical robot, keratoplasty, corneal transplantation, microfabrication

## Abstract

In penetrating keratoplasty (PKP), the proper corneal suture placement is very important for successful transplantation and restoring functional vision. Generating sutures with accurate depth is difficult for the surgeon because of the tissue’s softness, lack of depth information, and hand tremors. In this paper, an automatic cornea grasping device is proposed, which detects when the device reaches the target suture depth. When the device reaches the target depth, the device rapidly grasps the cornea to prevent error induced by human hand tremors. In the paper, the performance of the proposed sensor, the actuator, and the device are experimentally verified with ex vivo experiment. The result showed that the proposed device could enhance the accuracy and precision of the corneal suture depth.

## 1. Introduction

The cornea is the front part of the eye, which allows light to enter the eye. It is very soft, transparent, and has a thickness of about 550 μm. When the cornea is diseased or damaged irreversibly, a corneal transplant may be necessary to restore its function. Penetrating keratoplasty (PKP) is a full thickness transplant procedure that replaces a diseased cornea with the donor cornea, requiring a trephination of the patient’s cornea, followed by placement of a full-thickness donor corneal graft. In general, about sixteen interrupted sutures are placed radially around the edge of the cornea. Proper corneal suturing is needed to make an appropriate connection of the recipient with the donor cornea and avoid irregular surgical-induced astigmatism. If the suture depth is too thick, it can penetrate the cornea, which causes corneal endothelial cell loss. On the contrary, if the suture depth is too shallow, the anterior tissues of the recipient and the donor cornea cannot make appropriate wound strength. This incomplete wound construction causes wound leaks and irregular astigmatism [1,2,3].

To generate corneal suture in PKP, the surgeon typically uses two medical apparatus: forceps and suture needle. First, the surgeon inserts one tip of the forceps between the boundary of the patient eye and the donor cornea. The surgeon moves the forceps to locate the forceps’ tip at the target depth, which should fall just anterior to the Descemet’s membrane, which is about 90% of corneal thickness. When the surgeon thinks the forceps tip is located at the proper depth, the surgeon grasps the corneal using the forceps. A suture needle is inserted just below the forceps tip to generate a suture with target depth after grasping. Therefore, accurately grasping the cornea using forceps is key to proper suture placement.

However, grasping the cornea at target depth is a challenging task for surgeons. The difficulty is because of several reasons. First, transparency, softness, and thinness of the cornea make the surgeon challenging to figure out the current forceps depth. Second, a human’s natural hand tremor makes positioning of the forceps difficult. Even if the surgeon knows the forceps’ exact depth, positioning the forceps at that depth is challenging because of hand tremors. When humans hold the medical tool, the amplitude of hand tremors can be up to 200 μm [4]. The effect of hand tremor is critical in PKP because the corneal thickness is very thin, about 550 μm. As a consequence, only very skilled surgeons can perform accurate suturing in PKP.

To decrease the difficulty of corneal suturing, previous studies suggested several suturing-assistance devices. A device holding the cornea using negative pressure was suggested for more stable fixation of the corneal tissue [5]. A corneal suturing device integrated with an Optical Coherence Tomography probe was suggested to provide an image of the suture needle within the corneal tissue [6]. However, all the experiments were conducted with the uncut cornea, which is divided into the recipient-side cornea and the donor-side cornea in actual surgery.

Several robot systems were suggested for suturing in PKP. The feasibility of PKP using Da Vinci robot was shown [7,8]. However, there was no analysis of suture shape. Corneal grafting robot system having trephine device, needle gripper, and suture gripper were suggested, but only stitching force during suturing was measured as a suturing performance [9,10]. A robot system with a novel cornea gripper and needle insertion mechanism was suggested recently, which showed the possibility of controlling the suture depth [11].

This paper proposes a sensor-embedded automatic forceps, which detects the moment of reaching target suture depth and automatically grasps the cornea. The device is a hand-held type; therefore, we expect that the device has benefits of easy usage and a short learning curve compared to the robot system. The proposed device consists of two parts: a sensing part and an actuating part. The sensing part detects the moment of the device’s tip reaching the target depth. The actuation part automatically grasps the corneal tissue when the sensor is activated. The actuation part was designed to grasp the corneal as fast as possible, minimizing the effect of human hand tremors. The concept of the sensor-embedded forceps was firstly proposed in the previous conference paper [12]. However, the limitation was that there was no verification of the device’s suturing performance through experiment, and the device was not made of biocompatible material. The contributions of this paper are as follows. First, the device’s suturing performance was analyzed by conducting a suturing experiment on multiple porcine corneas. The device’s suturing performance is compared to that of the control group, suturing without the device. Second, the proposed device is made of biocompatible material. Therefore, the paper shows the possibility of using the device in actual surgery. In addition, the structure and the fabrication process of the device are fully introduced.

## 2. Materials and Methods

### 2.1. Automatic Grasping Mechanism

**Figure 1:**
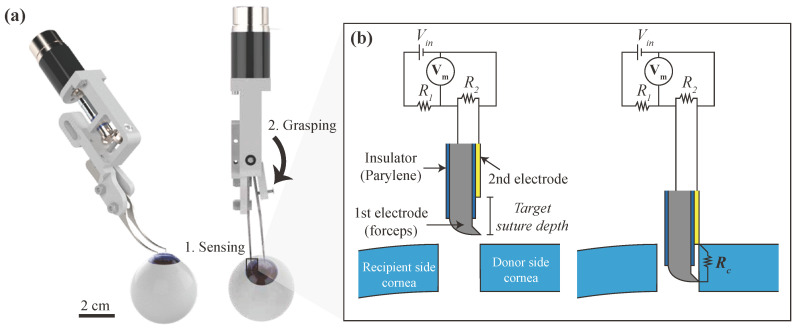
Mechanism of the proposed device. (**a**) When the device’s tip is inserted into the target suture depth, the sensor signal is activated, and the device rapidly grasps the cornea to prevent further position error induced by a human’s hand tremor. (**b**) The sensor part consists of two electrodes. When the device’s tip is inserted into the target suture depth, two electrodes are electrically connected through corneal tissue.

The mechanism of the device is shown in Figure 1. The device consists of two parts. The first is the sensing part, which detects the moment of the device reaching target depth. The second part is the actuation part which automatically grasps the cornea when the sensor signal is activated.

The sensor part is composed of two electrodes. The first electrode is the forceps body itself, which is stainless steel. The forceps body is coated with Parylene to insulate it from the second electrode. After insulation, Parylene at the tip of the forceps was selectively removed. The second electrode, a thin stainless steel sheet, is attached to the Parylene-coated forceps body’s surface. The two electrodes are electrically disconnected because of the insulator between them. The distance between the two electrodes is designed to be equal to the target suture depth.

To generate suture with the device, the surgeon slowly inserts the device’s tip between the recipient side cornea and the donor side cornea. When the device’s depth is less than the target suture depth, the two electrodes are electrically disconnected. When the device reaches the target suture depth, the corneal tissue is connected to both the first and second electrodes. Now the two electrodes are electrically connected through the corneal tissue. This electrical connection induces resistance to change in the detection circuit, and it can be detected by measuring a voltage $V_{m}$.

(1) $$V_{m}=V_{in}-R_{1}V_{in}{\left[R_{1}+\frac{R_{2}R_{c}}{R_{2}+R_{c}}\right]}^{-1}$$

(2) $$R_{c}=\left\{\begin{array}{cc} \infty , & \mathrm{if}\;d_{f}<d_{t}\\ R_{c}, & \mathrm{otherwise} \end{array}\right.$$

where $V_{m}$ is the voltage measured, $V_{in}$ is the voltage applied to the circuit, $R_{c}$ is the resistance of the corneal tissue, $d_{f}$ is current depth of the device’s tip, and $d_{t}$ is the target suture depth. Although the tissue’s electrical resistance $R_{c}$ is relatively high, the cornea has lower resistance than other tissues because of the eye fluid. The difference is enough to be measured by the circuit.

The proposed sensor has to be dried after grasping for the following other sutures. When the device grasps the corneal tissue, the sensor is wet because of the eye fluid. This eye fluid remains on the sensor even after the tissue is released. Therefore, the device should be dried before the next suture. The amount of remained eye fluid can be identified from the sensor signal. If eye fluid is thoroughly dried, the resistance between the electrodes will be infinite. In the experiments, we observed that eye fluid was thoroughly dried within dozens of seconds.

### 2.2. Sensor Fabrication

**Figure 2:**
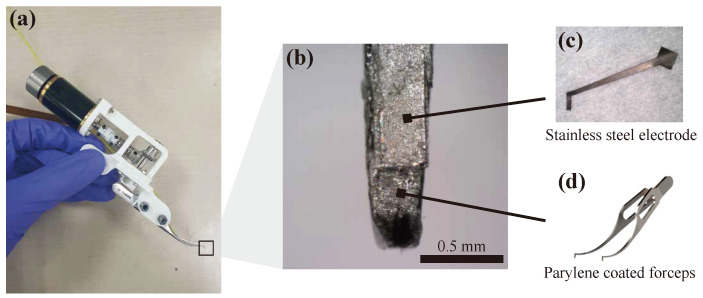
(**a**) Automatic grasping forceps consist of the sensor part and the actuation motor. (**b**) Fabricated sensor part. The first electrode is a metal forceps body itself. The surface of the forceps is coated with Parylene for insulation. The coating around the forceps tip is intentionally removed. The second electrode is made by cutting thin stainless steel sheet. The second electrode is attached to the surface of the forceps. (**c**) Second electrode made of stainless steel sheet. The large space in the upper right corner is for attaching electrodes. (**d**) CAD image of the forceps used in the device.

As the first electrode of the device, conductive medical toothed forceps for ophthalmic surgery, *Katena, k5-1500* is used. The forceps is made of stainless steel and has small teeth at its tip for preventing slipping. The forceps is Parylene-coated with 5 μm thickness. After the coating, the Parylene coating around the forceps tip is partially removed. Parylene is known as a biocompatible material, which is used in several medical applications, including ophthalmology [13,14]. The second electrode, which is attached to the forceps’ surface, is fabricated by cutting a thin stainless steel 316L sheet from Nilaco Corporation, Tokyo, Japan. The thickness of the stainless steel is 20 μm. The stainless steel sheet must be precisely cut to be attached to the complex-shaped forceps body. The stainless steel sheet is cut using a paper cutter Cameo 4 pro, Silhouette Korea, Seoul, Korea. The final stainless steel electrode is illustrated in Figure 2. The first and second electrodes are biocompatible because stainless steel is a well-known biocompatible material. The one end of the electrode is cut with relatively large space, and an electric wire is attached using silver epoxy. After the stainless steel electrode is fabricated, it is attached to the forceps body using epoxy. The distance between the first electrode and the second electrode is set to 500 μm.

### 2.3. Actuation Part

To automatically grasp the corneal tissue when the sensor signal is activated, the device is designed to transfer motor torque to the forceps. As a motor, Maxon, Sachseln, Switzerland, EC 20 flat (product number: 351006) with gear ratio 20:1 (product number: *134160*) is used. As a motor driver, Maxon, Sachseln, Switzerland, Epos 4 is used. The operating voltage of the system is set to 12 V. The motor is current-controlled to get the fastest grasping speed. The control law is as follows.

(3) $$I=\left\{\begin{array}{cc} I_{max}, & \mathrm{for}\;t<t_{1}\\ \frac{I_{max}-\left(I_{max}-I_{h}\right)\left(t-t_{1}\right)}{t_{2}-t_{1}}, & \mathrm{for}\;t_{1}<t<t_{2}\\ I_{h}, & \mathrm{for}\;t_{2}<t \end{array}\right.$$

where *I* is the motor current, $I_{max}$ is the target current during the grasping motion, $I_{h}$ is the holding current. *t* is the time after the sensor signal is activated, $t_{1}$ is the full-power grasping time, $t_{2}$ is the holding time. After the sensor signal is activated, the motor current is controlled to be $I_{max}$ to transfer maximum torque to the forceps. By applying maximum current, the fastest grasping speed can be achieved. After $t_{1}$, the motor current is decreased to $I_{h}$ at $t_{2}$. After $t_{2}$, the motor current is controlled to be $I_{h}$, which generates just enough torque to holds the corneal tissue. Decreasing motor current from $I_{max}$ to $I_{h}$ is to prevent damage of the corneal tissue due to high grasping force. In the experiments, $I_{max}$ was 1500 mA, $I_{h}$ was 900 mA, $t_{1}$ was 60 ms, and $t_{2}$ was 1000 ms.

During the grasping motion, excessive grasping force may damage the corneal tissue. The grasping force can be divided into two parts. The first is peak force that appears during fast grasping motion just after the motor is actuated. The second is settling force, which is a long period force after the motor is fully rotated. The proposed device prevents excessive peak force and settling force with two methods.

**Figure 3:**
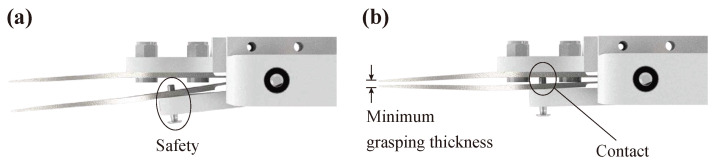
(**a**) Safety structure to prevent excessive grasping force. The bolt is assembled with the rotating part of the device. (**b**) When the device grasps the tissue, the grasping thickness is limited because the safety bolt contacts the opposite forceps body and prevents further rotation. The minimum grasping thickness can be precisely adjusted by turning the bolt.

Firstly, the device has a safety structure that limits the grasping thickness to prevent excessive peak force (Figure 3). The safety has a bolt whose length can be adjusted. When the maximum rotation angle is reached, the safety bolt’s tip contacts the opposite forceps body and prevents further grasping. Consequently, excessive grasping force can be prevented.

Secondly, the appropriate settling force was experimentally found to prevent tissue damage due to the high settling force. To find the appropriate force, we incrementally increased the motor current $I_{h}$ until the device stably grasps the tissue without slip. Consequently, the minimum grasping force without a slip of tissue could be found.

### 2.4. Measurement Circuit

An electrical circuit is composed to measure resistance between the first electrode and the second electrode. The diagram of the circuit is illustrated in Figure 1. The resistor $R_{1}$ with 5.1 MΩ and the $R_{2}$ with 200 kΩ were used in the experiments. The output voltage $V_{m}$ is measured using *Maxon, Epos4*, with 300 Hz of measurement frequency. The circuit voltage $V_{in}$ is set to 5 V before the grasping but changed to 0 V after the grasping.

(4) $$V_{m}=\left\{\begin{array}{cc} 5\;V, & \mathrm{if}\;V_{m}<V_{m0}+V_{th}\\ 0\;V, & \mathrm{otherwise}\; \end{array}\right.$$

where $V_{m0}$ is an initial voltage which is the average of the first fifty measurements of $V_{m}$. $V_{th}$ is a threshold voltage of 200 mV. After grasping the corneal tissue, the input voltage $V_{in}$ is lowered to zero to prevent corneal tissue damage due to electrical current.

### 2.5. Preparation of Corneal Specimen

In the experiments, porcine corneas enucleated from dead pigs were used as a specimen. All pigs were slaughtered for other purposes, not for the experiments. All porcine corneas were kept in a refrigerator before usage. All porcine corneas in the experiments were used within 12 h after enucleation. Before each experiment, the porcine cornea was cut into a circular shape with 8 mm diameter to be used as a donor cornea specimen. The remaining porcine eye was used as a recipient side eye specimen.

## 3. Results

### 3.1. Base Resistance and Resistance of Porcine Cornea

A base resistance of the device and a resistance of the porcine cornea are measured (Table 1). The resistance between the ground and the tip of the first electrode was 6.7 Ω. The resistance between the ground and the tip of the second electrode was 3.5 Ω. The total base resistance was 10.2 Ω. The system’s base resistance is very low; therefore, the base resistance is neglected in the circuit model and the experiment.

A resistance of eight porcine corneas was measured (Table 2). As a measurement probe, two electrodes of the devices were used. Therefore, the distance between probes was 500 μm. As a result, the mean resistance was 14.14 MΩ and the standard deviation was 13.1 MΩ. The standard deviation of the resistance was high compared to the mean value, and this is because the cornea’s resistance is highly dependent on the moisture condition of the corneal surface.

### 3.2. Sensor Signal While Linearly Moving Device’s Tip toward Corneal Tissue

The sensor signal was measured while moving the sensor-embedded device tip toward the corneal tissue. The device’s tip is inserted using 3-axis motorized stage from Shutter Instrument, Novato, USA, ROE-200. As a specimen, the porcine cornea was cut to 4 × 3 mm size with its thickness. Before inserting the device, the height of the device’s tip and the cornea’s surface was aligned. During the insertion, the output voltage $V_{m}$ was measured with 300 Hz measurement frequency. Unlike the later experiments, $V_{in}$ is maintained to 5 V for observing the sensor voltage $V_{m}$ during the whole insertion. In the later experiments, $V_{in}$ is decreased to zero, as explained in Equation (4).

**Figure 4:**
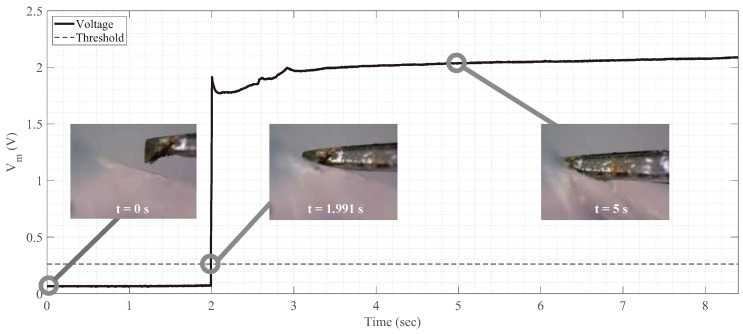
Sensor’s output voltage during linear insertion of the device toward the corneal tissue. Before *t* = 1.991 s, the voltage does not change even though the first electrode (forceps) touches the corneal tissue. At *t* = 1.991 s, both electrodes touch the corneal tissue, and the voltage rapidly increases.

The measured voltage $V_{m}$ during the insertion is illustrated in Figure 4. In the figure, insertion started at *t* = 0. The sensor voltage remained constant before the second electrode touches the corneal tissue. Between *t* = 0 s and *t* = 1.991 s, the first electrode is in contact with corneal tissue but there is no change of sensor voltage. At *t* = 1.991 s, the second electrode contacts corneal tissue, and the sensor voltage rapidly increases. After *t* = 1.991 s, both the first and the second electrode touch the corneal tissue, and sensor voltage remains high.

### 3.3. Measurement of Grasping Time

**Figure 5:**
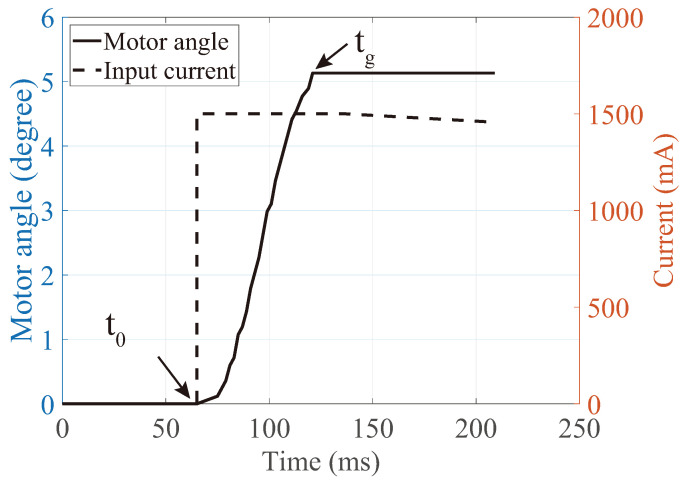
Angle of the motor during the grasping motion. The grasping was conducted without corneal tissue. The grasping time for full-grasp was 56 ms. With the corneal tissue, the grasping time is always shorter than 56 ms because the cornea’s thickness reduces the maximum grasping angle.

A grasping time, which is a duration between the start of torque generation and the full-grasping of forceps, is measured. The motor angle was measured using the motor’s hall sensor during the grasping motion without corneal tissue (Figure 5). In the figure, the grasping time can be defined as $t_{g}-t_{0}$, where $t_{0}$ is the moment of torque generation and $t_{g}$ is the moment of full grasping. The result shows that $t_{0}$ is 65 ms and $t_{g}$ is 121 ms. The grasping time, $t_{g}-t_{0}$, was 56 ms. The grasping time is always shorter than 56 ms with the corneal tissue because the cornea’s thickness reduces the maximum grasping angle.

### 3.4. Suturing on Porcine Cornea

**Figure 6:**
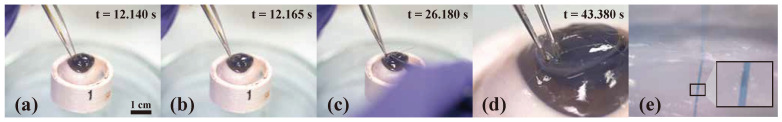
Suturing on the porcine cornea. (**a**) Just before the device’s tip reaches the target depth. (**b**) Just after the device’s tip reaches the target depth and the device grasp the cornea. (**c**) Needle insertion. (**d**) After pulling the needle, the suture is generated. (**e**) Microscopic image of the side surface of the corneal tissue. An exit point of the suture can be observed in the magnified image.

Porcine corneas were sutured using the proposed device, and the resulting suture depths were measured (Figure 6 and Video S1). The porcine cornea was put in the cornea holder, which is made by rapid prototyping. Total eight porcine corneas were used in the experiment. For each cornea, two sutures were made using the proposed automatic grasping device. On the same cornea, two sutures were made using traditional toothed forceps for comparison. Therefore, sixteen sutures were generated using the proposed device, and sixteen sutures were generated using the traditional toothed forceps. In the experiment, needle insertion was done manually using a needle holder from Rumex, Florida, USA, 8-024T. After suturing, the cornea is moved to the microscopic stand, and the suture depth was measured using microscope DinoLite, New Taipei City, Taiwan, AM7915MZT. All sutures were made on the donor side cornea to accurately move and image the cornea’s side surface.

**Figure 7:**
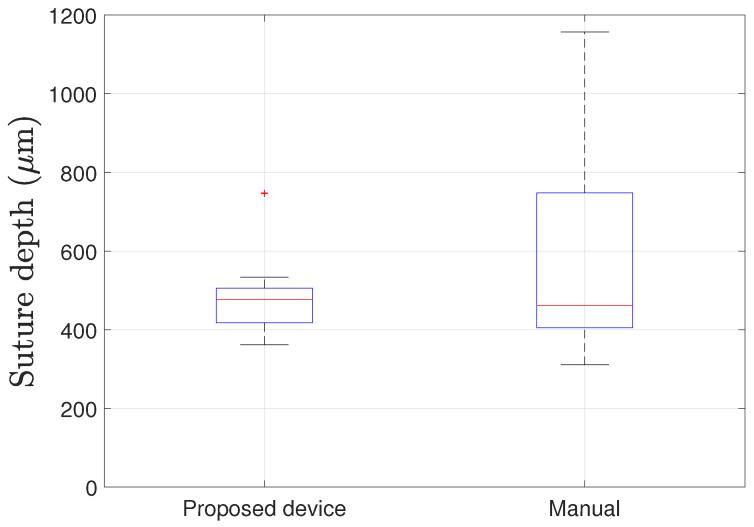
Box plot of the suture depth generated from the proposed device and manual suture. For each method, sixteen sutures were generated. With the device, there was one outlier (calculated with 1.5 IQR). Excluding the outlier, the median, maximum, and minimum suture depths were 477, 534, and 362 μm. Without the device, the median, maximum, and minimum suture depths were 461.5, 1157, and 311 μm.

The result of measuring suture depth is shown in Figure 7 and Table 3. The corneal thickness of 550 μm was assumed, and the target suture depth was designed to be 500 μm (90% of thickness). The mean suture depth generated using the proposed device was 447.1 μm, 81.3% of corneal thickness. This depth lies in the empirically reported safety boundary [15]. There may be several reasons why the depth is shallower than the target depth. First, the eye’s wet condition might connect the two electrodes before the device reaches the target depth. The device detects the moment of reaching the target depth by measuring the two electrodes’ electrical connections. When the second electrode is near the cornea’s top surface, moisture on the cornea can wet the second electrode. Since eye fluid is conductive, two electrodes will be electrically connected, and the device will misjudge the contact moment. The second reason for shallower depth might be an error induced by wrong needle insertion. After the device grasps the cornea, the operator manually inserts the needle just below the device tip. However, a human error can occur in this step which causes larger or smaller suture depth.

The standard deviation of the sutures from the proposed device was 85.8 μm, which is smaller than that from the manual suturing, 221.6 μm. There is an outlier in the result from the proposed device, which is 747 μm. The outlier can be because of two reasons. First, the device might not be inserted in contact with the cornea but suddenly come into contact with a deep insertion depth. The device’s surface must contact the cornea from the beginning of the insertion to properly detect the insertion depth. If not, the second electrode’s first contact with the corneal tissue can happen deeper. The second reason for the outlier is wrong needle insertion after the grasping. The operator manually inserts the needle, and this can induce an error in the suture depth.

## 4. Discussion

Accurate and precise suturing in PKP is a challenging task, which can cause improper wound construction, and even additional surgical procedures or regraft may be required. The difficulty mainly comes from lack of depth information and human hand tremor. This paper proposed an automatic cornea grasping device that detects the device’s moment reaching the target depth and rapidly grasps the corneal tissue to minimize error from hand tremors. The performance of the sensor and the actuator was verified through the experiment. Sutures generated using the device and sutures made manually were compared. The result showed that the proposed device could enhance the accuracy and precision of the suture depth.

The contributions of this work are as following: First, the automatic grasping device is proposed, which can enhance the corneal suture depth’s accuracy and precision. The device is hand-held type; therefore, easy usage and a short learning curve are expected as benefits. Second, all component contact with corneal tissue is made of a biocompatible material, and a detailed fabrication process is explained. Third, the performance of the proposed sensor and actuator were experimentally verified. Forth, the suturing performance of the device was experimentally verified using porcine corneas.

As a result, the sutures generated by the proposed device showed a shallower depth than the target depth. Shallower depth may occur because of the corneal surface’s wet condition, which comes into contact with the device’s electrode before reaching the target depth. To prevent this, avoiding the hydration on the corneal surface around the suture position needs to be added to the current protocol.

The precision of the suture depth was enhanced compared to the manual suture by using the proposed device. However, there was the outlier in the result. The outlier might be the no contact between the device and the cornea after the device insertion. To prevent this, the operator should pay more attention to maintaining contact between the device and the cornea from the beginning of the device insertion. Moreover, manual needle insertion can induce suture depth error. To prevent this, attaching an additional needle insertion device, such as in [11], can be considered in future work.

## Figures and Tables

**Table 1:** Base resistance of the proposed device.

Ground to 1st Electrode	Ground to 2nd Electrode	Total (1st + 2nd Electrodes)
6.7 Ω	3.5 Ω	10.2 Ω

**Table 2:** Resistance of porcine cornea, measured using two electrodes of the device as measurement probes.

Resistance of Porcine Cornea (MΩ)									
Eye 1	Eye 2	Eye 3	Eye 4	Eye 5	Eye 6	Eye 7	Eye 8	Mean	Std.
3.7	5.8	13.8	5.3	6.4	5.5	36.3	36.3	14.1	13.1

**Table 3:** Measured suture depth.

Proposed Device		Manual	
Mean (μm)	Std. (μm)	Mean (μm)	Std. (μm)
447.1	85.8	573.3	221.6

## Data Availability

The data presented in this study are available on request from the corresponding author.

## Supplementary Materials

The following is available online at https://www.mdpi.com/article/10.3390/mi12050484/s1, Video S1: Suturing on the porcine cornea using the proposed device.
